# Cognitive Behavioral Therapy in Psychiatric Nursing in Japan

**DOI:** 10.1155/2015/529107

**Published:** 2015-12-20

**Authors:** Naoki Yoshinaga, Akiko Nosaki, Yuta Hayashi, Hiroki Tanoue, Eiji Shimizu, Hiroko Kunikata, Yoshie Okada, Yuko Shiraishi

**Affiliations:** ^1^Organization for Promotion of Tenure Track, University of Miyazaki, 5200 Kihara, Kiyotake, Miyazaki, Miyazaki Prefecture 889-1692, Japan; ^2^Division of Psychiatric and Mental Health Nursing, Graduate School of Nursing, Chiba University, 1-8-1 Inohana, Chuo-ku, Chiba, Chiba Prefecture 260-8672, Japan; ^3^Inpatient Psychiatric Unit, Chiba University Hospital, 1-8-1 Inohana, Chuo-ku, Chiba, Chiba Prefecture 260-8677, Japan; ^4^Department of Psychiatric and Mental Health Nursing, Graduate School of Nursing, University of Miyazaki, 5200 Kihara, Kiyotake, Miyazaki, Miyazaki Prefecture 889-1692, Japan; ^5^Department of Cognitive Behavioral Physiology, Graduate School of Medicine, Chiba University, 1-8-1 Inohana, Chuo-ku, Chiba, Chiba Prefecture 260-8670, Japan; ^6^Department of Nursing, Faculty of Health Sciences, Kagawa Prefectural University of Health Sciences, 281-2 Murechohara, Takamatsu, Kagawa Prefecture 761-0123, Japan; ^7^Department of Mental Health and Psychiatric Nursing, Graduate School of Comprehensive Human Sciences, University of Tsukuba, 1-1-1 Tennodai, Tsukuba, Ibaraki Prefecture 305-8577, Japan

## Abstract

Psychiatric nurses have played a significant role in disseminating cognitive behavioral therapy (CBT) in Western countries; however, in Japan, the application, practice, efficiency, and quality control of CBT in the psychiatric nursing field are unclear. This study conducted a literature review to assess the current status of CBT practice and research in psychiatric nursing in Japan. Three English databases (MEDLINE, CINAHL, and PsycINFO) and two Japanese databases (Ichushi-Web and CiNii) were searched with predetermined keywords. Fifty-five articles met eligibility criteria: 46 case studies and 9 comparative studies. It was found that CBT took place primarily in inpatient settings and targeted schizophrenia and mood disorders. Although there were only a few comparative studies, each concluded that CBT was effective. However, CBT recipients and outcome measures were diverse, and nurses were not the only CBT practitioners in most reports. Only a few articles included the description of CBT training and supervision. This literature review clarified the current status of CBT in psychiatric nursing in Japan and identified important implications for future practice and research: performing CBT in a variety of settings and for a wide range of psychiatric disorders, conducting randomized controlled trials, and establishing pre- and postqualification training system.

## 1. Introduction

Mental health treatments that are both effective and accessible to the general population are in high demand. In the field of psychiatry, cognitive behavioral therapy (CBT) has been widely practiced for over 40 years in Western countries. CBT is a time-limited, present-focused, and goal-oriented psychotherapy that helps patients learn and apply specific strategies to modify cognitions and behaviors in their own environment through homework [1]. There has been a rapid accumulation of evidence supporting its efficacy for a wide range of mental disorders [2].

Psychiatric nurses have played a significant role in disseminating this psychotherapy, especially in UK, and numerous clinical trials have studied the effects of CBT conducted by psychiatric nurses [3–5]. In the 1970s, psychiatric nurses became the first group outside psychiatrists or psychologists to receive systematic CBT training at Maudsley Hospital in London. Further, a 25-year follow-up of nurses administering CBT found a considerable contribution to mental health service provision, specifically in primary care settings [4]. In addition, CBT practiced by psychiatric nurses has been found to be cost effective [6, 7], and, as far as we are aware, no research knows that one mental-health professional has greater clinical efficacy than any other one.

In Japan, CBT was introduced in the late 1980s; the number of research articles and case studies about CBT has increased since 1989 [8]. Approximately 10 years later, CBT began to be used by nurses [9]. In April 2010, the inclusion of CBT for mood disorders in the national health insurance system marked a milestone for psychiatric care and interventions in Japan, where pharmacotherapy has traditionally been much more common. However, access to CBT services is extremely limited due to an insufficient number of CBT providers in the current health insurance system, which requires CBT to be conducted by skilled psychiatrists [10]. To solve this problem, expanding the range of CBT providers to include other medical staff, particularly psychiatric nurses, has been proposed because the qualification for psychologists is not a national qualification (i.e., private-sector qualification) in Japan [11]. However, the existing literature about CBT practice and research in Japan does not focus on nurses (e.g., about the effectiveness of CBT provided by psychiatrists and psychologists) [12, 13] and therefore the application, practice, efficiency, and quality control of CBT in psychiatric nursing are unclear.

In this study, a literature review was conducted to assess the current status of CBT practice and research in the field of psychiatric nursing in Japan. To achieve this objective, we identified the formats and settings, recipients, effectiveness of the treatment, and the training and supervision received.

## 2. Materials and Methods

Research articles were included in this review if nurses provided CBT for patients with psychiatric disorders in Japan. Both case studies and comparative studies were included in this review. Articles were primarily identified through the following electronic databases: MEDLINE (1950–), CINAHL (1937–), and PsycINFO (1806–) for the English literature and Ichushi-Web (1983–) and CiNii (1946–) for the Japanese literature. The keywords used, in English and Japanese, were “nurse^*∗*^ OR nursing (*kango*)” and “behavio^*∗*^ therapy (*koudou-ryouhou*) OR cognitive therapy (*ninchi-ryouhou*) OR cognitive behavior^*∗*^ therapy (*ninchi-koudou-ryouhou*)” and “Japan^*∗*^.” The final search was performed on 5 November 2015, and the search strategy is detailed in Supplementary Appendix 1 in Supplementary Material available online at http://dx.doi.org/10.1155/2015/529107. In addition to searches of the online databases, hand searches were performed by scanning the reference lists of those papers identified by the database searches. Experts in the field were also consulted to identify any existing research.

The identified articles were subject to the following inclusion and exclusion criteria. Inclusion criteria consisted of the following: (1) a nurse is the CBT provider or member of the CBT providing team (a facilitator was included if the article reported a group therapy format), (2) the targeted patients were diagnosed with psychiatric disorders, and (3) the study included an intervention (case study or comparative study). Exclusion criteria included (1) reviews or commentaries, (2) retrospective analyses carried out using only CBT theory, (3) secondary analyses of a published research, and (4) reports of less than two pages that did not include a detailed description of how quality of CBT was ensured.

Initial literature search was performed by a member of the research team (Naoki Yoshinaga). Eligibility assessment and data abstraction were performed collaboratively by three reviewers (Naoki Yoshinaga, Akiko Nosaki, and Yuta Hayashi). Abstracted data included eligibility criteria, formats and settings, recipients, effectiveness of the treatment, and the training and supervision received. Disagreements between reviewers were resolved by consensus. The title and the abstract for all records were read to increase detection accuracy. This method of record assessment has been shown to increase the accuracy of selecting relevant papers without increasing the inclusion of the irrelevant literature [14]. Furthermore, the selected literature was categorized as either “comparative study,” for intervention studies that included quantitative analysis, or as a “case study.”

## 3. Results

**Figure 1:**
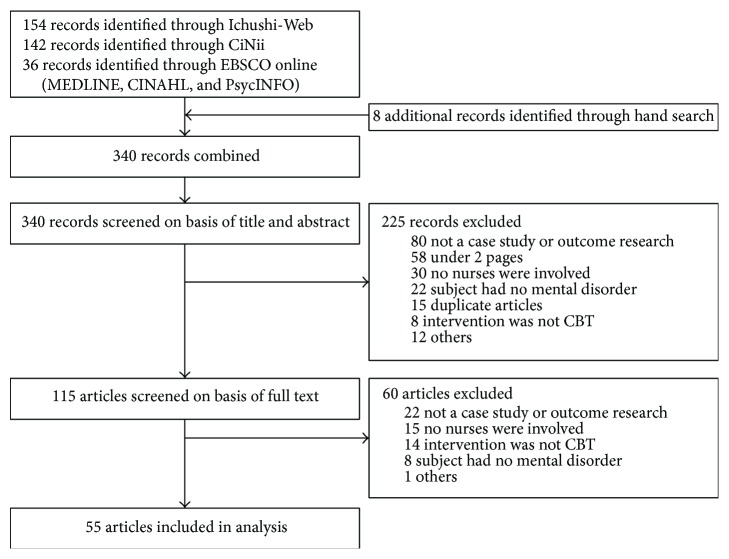
Literature selection process.

The selection process is shown in Figure 1. Using the search strategy, 332 articles were retrieved from the databases and additional 8 articles identified by hand searches (total 340), of which 225 were excluded on the basis of the title and abstract. Of the remaining 115 articles, 60 were excluded after reading full manuscripts. The remaining articles (*n* = 55) met eligibility criteria. Of these 55 studies, 46 were case studies (see Supplementary Appendix 2 for all case study references) and 9 were comparative studies.

### 3.1. Format and Setting

In the case studies (Table 1), inpatient settings were the primary location of CBT. In about half of the studies (*n* = 25) the format was “unknown/not described”; when it was described, individual format was the most common format (*n* = 17), followed by group format (*n* = 4).

In the comparative studies (Table 1), the study design employed was predominantly single-arm trials, and two studies used a randomized controlled trial design [15, 16]. The setting was inpatient (*n* = 4), outpatient (*n* = 4), and community setting (*n* = 1). In the studies using individual therapy, there were no studies in which a nurse was solely responsible for structuring the CBT course of treatment; doctors, clinical psychologists, psychiatric social workers, and pharmacists were also involved. In the studies reporting group therapy, the nurse provided CBT in three studies [15, 17, 18], and the remainder of articles involved a number of other clinicians, such as psychiatrists and clinical psychologists. In case of involvement of the other disciplines, nurses provided CBT as “subtrainer” and “observer.” But there is no detailed description about the role of the nurse in the articles.

### 3.2. CBT Recipients

In the case studies, the subjects receiving CBT were predominantly patients with schizophrenia, followed by patients with mood disorders and developmental disorders.

Contrary to the case studies, in the comparative study, the subjects were primarily patients with mood disorders, followed by patients with schizophrenia and with anxiety disorders (Table 1).

### 3.3. CBT Effectiveness in Comparative Studies

The details of the comparative studies are shown in Table 2. As stated before, there were only three studies where nurses were solely responsible for structuring treatment [15, 17, 18]. Of those, two studies used a single-arm trial [17, 18] and targeted female patients living with depression and people living in the general community with mental disabilities (schizophrenia and mood disorder), all of whom received group therapy. Both studies reported that group CBT effectively reduced psychiatric symptoms after intervention. One study [15] used a randomized controlled trial, where inpatients with schizophrenia received group therapy. As a result, the intervention group, which received group CBT, had greater knowledge of disease self-management and improved in speech skill, and social activity score compared to a control group. In other comparative studies, in which therapists were not solely psychiatric nurses, CBT was also reported to be effective.

Overall, in the comparative studies, CBT recipients and outcome measures were widely diverse, and the pre- and posteffect sizes (Cohen's* d*) of CBT arm were ranged from 0.29 (small) to 1.79 (large).

### 3.4. CBT Training and Supervision

Descriptions of the quality of CBT were divided into “therapist background and training” and “quality control and evaluation of CBT techniques” (Table 3).

A large majority (75%) of the studies did not describe the therapists' background and training (Table 3). In those that contained this information, the therapists received “closed (only study therapists can attend), short-term training courses directed by an expert (*n* = 8)” and others received “long-term training courses directed by an expert (*n* = 3),” “participation in voluntary study meetings with no expert present (*n* = 3),” “qualification acquisition related to CBT (*n* = 2),” and “open (anyone can attend), short-term workshop with experts (*n* = 2).”

Few studies described quality control or evaluation of the CBT techniques (Table 3). Reported quality control (methods to enhance CBT adherence) were “group supervision,” “individual supervision,” and “both group supervision and individual supervision.” Only two studies described the quality of CBT techniques; both used the Cognitive Therapy Scale Revised (CTS-R) to assess the quality of CBT techniques by reviewing videotaped sessions [19, 20].

## 4. Discussion

This is the first review of the existing literature to assess and summarize the current status of CBT practice and research in the field of psychiatric nursing in Japan. The main findings are fourfold.

First, nurses conduct CBT primarily in inpatient settings (especially in the case studies), whereas psychiatrist and psychologist provide CBT mainly in outpatient settings in Japan [10, 12]. A possible reason relates to the role and responsibilities of psychiatric nurses; they are able to perform physical and medication management and have frequent contact with patient by virtue of their 24-hour presence [24] and play an important role in the treatment of patients with severe psychiatric conditions in the inpatient setting. Moreover, the inpatient setting is a primary location for psychiatric practice in Japan because the length of psychiatric hospitalization is prolonged compared to other countries [25]. In Western countries, nurse-led CBT is applied in primary care and community settings rather than inpatient settings [4, 26]. For example, community psychiatric nurses delivered brief CBT for patients with schizophrenia [27] and for patients who repeatedly attempted suicide in community settings [28]. In our review, only three studies reported the use of CBT in the community/home-visit setting [18, 29, 30]; therefore, the setting for CBT is limited for psychiatric nurses in Japan.

Second, CBT provided by psychiatric nurses primarily targeted patients with schizophrenia and mood disorders. On the other hand, other mental professionals (psychiatrists and psychologists) in Japan and the Western countries provide CBT mainly for anxiety disorders [4, 13]. In UK, psychiatric nurses were initially trained to become therapists primarily for neurotic disorders such as specific phobia, obsessive-compulsive disorder, social phobia, and agoraphobia [4, 31]. In Japan, schizophrenia and mood disorders are the most commonly seen psychiatric disorders in both outpatient and inpatient settings; however, patients with anxiety disorders are commonly seen in an outpatient setting, not in inpatient settings [32]. Therefore, it seems that the disorders treated by psychiatric nurses using CBT may be strongly affected by the setting (CBT in nursing filed was conducted mainly in inpatient setting).

Third, the comparative studies (mostly before and after design) show indeed improvements after CBT. However, because of the lack of a control group, they cannot “prove” the effectiveness of CBT. Further, CBT therapists were not only nurses in most comparative study, and the CBT recipients and outcome measures were diverse. These factors do not allow for meta-analysis or the comparison of effectiveness across countries.

Finally, most studies did not include the description of CBT training and supervision. In Western countries, both basic training and clinical supervision are the primary methods used to develop and maintain competence in CBT, and there is some evidence that training can enhance therapists' competence and/or patient outcomes [33–35]. In Japan, the opportunities for nurses, psychiatrists, and psychologists to learn CBT have been gradually increasing through workshops held during annual conferences, and several institutions have begun to regularly provide a series of workshops. However, only a limited number of clinicians can receive such training because it is primarily offered in major urban areas, such as Tokyo. Moreover, on-going supervision is not available for most clinicians because the training is mostly performed on a one-off basis not on a continual basis. Therefore, it seems that the scarcity of research detailing CBT training and supervision is reflected by the realities of limited opportunities for receiving CBT training and supervision in Japan.

The current review has a number of limitations. First, given the relatively small number of studies, many conclusions could not be drawn. Second, the diversity of CBT recipients and outcome measures did not allow for an accurate assessment of CBT effectiveness in many domains. Third, some studies did not identify the primary outcome and measured many outcome variables; multiple comparison problem needs attention. Further, the usual methodology for systematic reviews was not followed in all respects, and this may weaken the conclusions of the study.

This survey of the existing literature clarified the current status of CBT in psychiatric nursing in Japan and provides important implications for future practice and research aimed at assessing aspects of CBT nursing in Japan. First, Japanese psychiatric nurses should consider performing CBT in a variety of settings and for a wide range of psychiatric disorders because CBT currently took place mainly in inpatient settings and targeted schizophrenia and mood disorders (few for anxiety disorder). Second, conducting randomized controlled trials that evaluate the effectiveness of CBT performed by nurses is required. As proposed by Gunter and Whittal [36], the effective dissemination of CBT requires clinical trials and the evaluation of empirical data to acquire public funding and organizational support. In addition to the need to accumulate evidence of the effectiveness of nurse-led CBT, a pre- and postqualification CBT training system should be established in each region of the country to allow more health professionals to receive adequate CBT training and on-going supervision. Recently, the Graduate School of Medicine at Chiba University set up a 2-year CBT training course for mental health professionals in 2010, the first postqualification course for CBT in Japan [19]. In addition, the Society for Research on CBT for Nurses recently established an experimental education program, special CBT training only for nurses that also includes on-going supervision [37]. Other than face-to-face training and supervision, online training/supervision can be an alternative method especially for professionals who live in a place where competent CBT therapists/supervisors are in short supply or nonexistent.

## 5. Conclusions

This literature review clarified the current status of CBT in psychiatric nursing in Japan and also identified important implications for future research aimed at assessing aspects of CBT practiced by psychiatric nurses in Japan.

## Supplementary Material

Supplementary Material provides the search strategy (Appendix 1) and all case study references (Appendix 2).

## Figures and Tables

**Table 1:** Summary of the case studies and comparative studies.

Type of study	Variable	*n*	(%)
Case studies (*n* = 46)	Setting		
	Hospital: inpatient care	40	(87.0)
	Hospital: outpatient care	4	(8.7)
	Others^§^	2	(4.3)
	Format		
	Individual	17	(37.0)
	Group	4	(8.7)
	Unknown/not described	25	(54.3)
	Recipient^†^		
	Schizophrenia	17	(37.0)
	Mood disorders	7	(15.2)
	Developmental disorders	5	(10.9)
	Obsessive-compulsive disorders	4	(8.7)
	Anorexia	3	(6.5)
	Social anxiety disorders	2	(4.3)
	Others	8	(17.4)

Comparative studies (*n* = 9)	Design		
	Single-arm trial	7	(77.8)
	Randomized controlled trial	2	(22.2)
	Setting		
	Hospital: inpatient care	4	(44.4)
	Hospital: outpatient care	4	(44.4)
	Others^¶^	1	(11.1)
	Format and therapists		
	Individual		
	NS, DR	1	(11.1)
	NS, DR, CP, PSW	1	(11.1)
	NS, DR, CP, PSW, PH	1	(11.1)
	Group		
	NS only	3	(33.3)
	NS, DR	1	(11.1)
	NS, CP	2	(22.2)
	Recipient^†‡^		
	Mood disorders	5	(55.6)
	Schizophrenia	2	(22.2)
	Anxiety disorders	2	(22.2)

^†^Primary diagnosis, ^‡^majority diagnosis if patients with a variety of diagnoses were recruited in the study, ^§^home visiting care, and ^¶^community care (program was held at community center). NS, nurse; DR, doctor; CP, clinical psychologist; PSW, psychiatric social worker; PH, pharmacist.

**Table 2:** Details of comparative studies (9 cases).

Study	Methods			Results	Effect sizes^†^
	Subjects	Therapist(s)	Design		
Kobori et al. (2014) [19]	Patients with anxiety disorders (obsessive-compulsive disorder, anorexia, and social anxiety disorder)	3 nurses; 3 doctors; 1 pharmacist; 2 psychiatric social workers; 13 clinical psychologists	Single-arm trial (*n* = 45); 12 weekly sessions of 50-minute individual CBT	Symptoms of depression (PHQ-9) and anxiety (GAD-7) improved significantly after intervention (*p* < 0.05)	Moderate effect on PHQ-9 (*d* = 0.68, 95% CI −0.08 to 1.39) and GAD-7 (*d* = 0.75, 95% CI −0.01 to 1.46)

Okada (2013) [17]	Female patients with unipolar depression	1-2 nurses	Single-arm trial (*n* = 78); 8 weekly sessions of 90-minute group CBT	Depressive symptoms (BDI-II) improvement after intervention (*p* < 0.05); improvement in cognitive impairment (DAS24) within 6 months of completion of intervention (*p* < 0.05); improvement in cognition and behavior in everyday life and with respect to other significant ones (interview data)	Moderate effect on BDI-II (*d* = 0.51, 95% CI −0.23 to 1.22)

Kunikata (2013) [18]	Persons with psychiatric illnesses living in the community (schizophrenia and mood disorder)	1 director (nurse); 1 facilitator (nurse)	Single-arm trial (*n* = 6); 12 biweekly sessions of 120-minute group CBT	No significant changes in self-esteem (RSES) or mood status (POMS) before and after intervention; mental sense of control (WHO-SUBI subscale) and psychiatric symptoms (BPRS) showing improvement before and after intervention (*p* < 0.05)	Moderate effect on RSES (*d* = 0.65, 95% CI −1.36 to 0.10)

Yoshinaga et al. (2013) [20]	Patients with social anxiety disorder	1 nurse; 1 doctor; 3 clinical psychologists; 1 psychiatric social worker	Single-arm trial (*n* = 15); 14 weekly sessions of 90-minute individual CBT	Social anxiety symptoms (LSAS, SPS, SIAS, FQ-SP, SFNE) showed improvement during and at the end of intervention (*p* < 0.05); after intervention, 73% of participants were judged to be treatment responders and 40% met the criteria for remission	Large effect on LSAS (*d* = 1.56, 95% CI 0.70 to 2.33)

Sakano et al. (2010) [21]	Inpatients with major depressive disorder and the related depressive symptoms	1 trainer (psychologist); 1-2 subtrainers (psychologist and nurse)	Single-arm trial (*n* = 54); 5 weekly sessions of 60-minute group CBT (for improving adequate emotional expression and interpersonal skills, inhibition of aggression, and preventing depression)	Depressive symptoms (BDI), social interaction anxiety (SIAS), social skills (SSS), fear of negative evaluation (SFNE), and QOL (WHOQOL-26) all showed improvement after the intervention (*p* < 0.05). In follow-up, improvement of fear of negative evaluation (SFNE) was maintained; long-term maintenance of other improvements was not observed	Moderate effect on BDI (*d* = 0.58, 95% CI −0.16 to 1.30)

Sakano et al. (2010) [22]	Inpatients with major depressive disorder and the related depressive symptoms	1 trainer (psychologist); 1-2 subtrainers (psychologist and nurse)	Single-arm trial (*n* = 62); 5 weekly sessions of 60-minute group CBT (for improving stress coping and depression)	After the intervention, anxiety and depressive symptoms (BDI, SRS-18 sub-items), lethargy (SRS-18 sub-items), and QOL (WHOQOL-26) showed improvement (*p* < 0.05); additionally, diversification of stress coping strategies (TAC-24) and increased ability to control stress (CARS) were observed (*p* < 0.05); however, long-term effects were not observed	Small effect on BDI (*d* = 0.29, 95% CI −0.44 to 1.00)

Watanabe et al. (2011) [16]	Patients with residual depression and refractory insomnia	5 doctors; 1 nurse	Randomized controlled trial; intervention group (*n* = 20) received usual care + 4 weekly sessions of 40-minute individual CBT; control group (*n* = 17) received only usual care	Compared to the control group, the intervention group's insomnia (ISI) and depressive symptoms (GRID-HAMD) had improved (*p* < 0.05)	Large effect on ISI (*d* = 1.79, 95% CI 0.90 to 2.58)

Kumagai et al. (2003) [15]	Hospitalized patients with schizophrenia	1 nurse	Randomized controlled trial; intervention group (*n* = 16) received group occupational therapy + 16 twice-weekly sessions of 120-minute group CBT; control group (*n* = 15) received only group occupational therapy	Compared to the control group, the intervention group had improved knowledge of disease self-management, speech skill, and social activity score (REHAB sub-items); there was no significant difference in QOL (WHOQOL-26) between the two groups	Moderate effect on DS score of REHAB (*d* = 0.63, 95% CI −0.12 to 1.34)

Okuno et al. (2000) [23]	Elderly patients with depression	2 doctors; 1 nurse (observer)	Single-arm trial (*n* = 18); 8 weekly sessions of 60-minute group CBT	After the intervention, 7 patients (39%) showed improvement (BDI reduction rate > 50%); regarding BDI subscale, loss of interest/pleasure and hypochondriac showed significant improvement (*p* < 0.01), and suppression, depressed mood, self-denial, and physical symptoms, also significantly improved (*p* < 0.05)	Large effect on BDI (*d* = 1.04, 95% CI 0.25 to 1.77)

^†^Pre- to posteffect sizes (Cohen's *d*) of CBT arm for each study were recalculated using same formula. BDI-II, Beck Depression Inventory-II; BPRS, Brief Psychiatric Rating Scale; CARS, Cognitive Appraisal Rating Scale; CBT, Cognitive Behavioral Therapy; DAS24, Dysfunctional Attitude Scale-24; FQ-SP, Fear Questionnaire-Social Phobia Subscale; GAD-7, Generalized Anxiety Disorder-7; GRID-HAMD, GRID-Hamilton Depression Rating Scale; ISI, Insomnia Severity Index; LSAS, Liebowitz Social Anxiety Scale; PHQ-9, Patient Health Questionnaire-9; POMS, Profile of Mood States; REHAB, Rehabilitation Evaluation of Hall and Baker; RSES, Rosenberg Self-Esteem Scale; SFNE, Short Form Fear Of Negative Evaluation; SIAS, Social Interaction Anxiety Scale; SPS, Social Phobia Scale; SRS-18, Stress Response Scale; SSS, Social Skills Scale; TAC-24, Tri-axial Coping Scale-24; WHO-SUBI, WHO-Subjective Well-Being Inventory; WHOQOL-26, WHO Quality of Life-26.

**Table 3:** Reporting on quality of cognitive behavioral therapy (*n* = 55).

	*n*	(%)
*Therapist background and training*		
Included description		
CBT training^†^		
Received closed, short-term training run by an expert	8	(14.5)
Received long-term training course run by an expert	3	(5.5)
Received voluntary study meetings with no expert	3	(5.5)
Qualification acquisition related to CBT	2	(3.6)
Received open, short-term workshop with experts	2	(3.6)
(Total)	17	(30.9)
CBT experience (total)	4	(7.3)
Lacked description (total)	41	(74.5)

*Quality control and evaluation of CBT techniques*		
Included description		
Supervision		
Received group supervision	3	(5.5)
Received individual supervision	3	(5.5)
Received both individual and group supervision	2	(3.6)
(Total)	8	(14.5)
Measures of CBT competence (total)	2	(3.6)
Lacked description (total)	47	(85.5)

^†^Includes duplicates. CBT, cognitive behavioral therapy.
