# Supplementary material for: Cognitive Behavioral Therapy in Psychiatric Nursing in Japan
Source: Nurs Res Pract. 2015 Dec 20;2015:529107. doi: 10.1155/2015/529107 (PMC4698669; doi:10.1155/2015/529107)
Supplement: Supplementary file 1 — Supplementary Material provides the search strategy (Appendix 1) and all case study references (Appendix 2). [file 529107.f1.pdf]

**Supplementary Appendix 1:** Search Strategy

Database: *EBSCOhost (MEDLINE, CINAHL, and PsychINFO)*

S1: nurse* OR nursing

S2: “behavio* therapy” OR “cognitive therapy” OR “cognitive behavior* therapy”

S3: Japan*

S4: S1 AND S2 AND S3

Database: *Ichushi Web*

#1: kango/TA

#2: koudou-ryouhou/TA or ninchi-ryouhou/TA or ninchi-koudou-ryouhou/TA

#3: #1 and #2

Database: *CiNii*

SERACH: kango AND (koudou-ryouhou OR ninchi-ryouhou OR ninchi-koudou-ryouhou)

**Supplementary Appendix 2:** Case Study References


Abe F, Takahashi A, Yoshino M, Takeda, N. Koudou ryouhou ga soukoushita batsumousho no kango. *Shoni Kango* 1988; **11**(9): 1051–1057 (in Japanese).

Bando, H. & Morita, N. Taijin fuan wo kakaeru kouhannsei hattatsu syougaisha eno kakawari: kateinai bouryoku wo okoshita kannja eno komyunikeishon shien. *Jpn. J. Psychiatr. Nurs. Soc.* 2008; **51**(2): 62–66 (in Japanese).

Fukagai C, Ohnishi R, Niimi, K. Seishinka heisakankyou ni okeru metaborikku shindoroumu no yobou/kaizen ni muketa ninchi koudou ryouhou no kouka: kanja no serufu efikashii no koujou to kenkouishiki no henyou wo mezashite. *Jpn. J. Psychiatr. Nurs. Soc.* 2009; **52**(2): 419–423 (in Japanese).

Fukuchi Y, Saito T, Ueki J. Taiou ni kuryoshita hi-gyakutaiji no ichiijirei. *Proc. Nurs. Res. Kanagawa Child. Med. Cent.* 2006; **29**: 60–62 (in Japanese).

Furuya K, Saito E. Tougou-shitchousho ni taisuru ninchi koudou ryouhou no kokoromi: shojo jikokanri gurupu to kobetsu kango no jissen. *Jpn. J. Psychiatr. Nurs. Soc.* 2010; **53**(3): 95–99 (in Japanese).

Hashimoto Y. Koudou ryouhou-teki apurouchi wo tooshite no kyouhaku shinkeisho kanja eno kango kara. *Jpn. J. Psychiatr. Nurs. Soc.* 1999; **42**(1): 548–550 (in Japanese).

Hata M. Shinkeisei shokuifushinsho no kango ni koudou ryouhou wo toriirete. *Iryo* 1984; **38**(3): 612–618 (in Japanese).

Hidaka F. Shinkeisei shokuifushinsho no kanngo: koudou ryouhou ni yotte taijuuzouka ga mirareta ichishourei. *Seishin Kango* 1986; **31**: 43–46 (in Japanese).

Hitomi T, Hattori M, Takeuchi A. Seinenki niaru mizuchuudoku kanja no insuiryou no ishikizuke ni sanyogata kango wo dounyuu shite: insui jikokanri ga dekita ichijirei no kousatsu. *Jpn. J. Psychiatr. Nurs. Soc.* 2007; **50**(2): 38–42 (in Japanese).

Inoue N, Mukaihara S. Byouteki tainsuikeikou ni taisuru ninchi koudou ryouhou wo mochiita apurouchi: chiteki syougai kanja no kainsui ga keigenshita jirei yori. *Jpn. J. Psychiatr. Nurs. Soc.* 2010; **53**(3) 90–94 (in Japanese).

Inoue N, Nakanishi Y, Honjo S. Kyoukaisei paasonaritii shougaisha ni taisuru bensyouhou-teki koudou ryouhou wo mochiita kango mensetsu no jisshi to sono kouka. *Proc. Jpn. Nurs. Assoc. (Psychiatr. Nurs.)* 2012; **42**: 64–67 (in Japanese).

Kanemaru N. Jishou koui wo kurikaesu kanja no ninchi eno kakawari wo kokoromite. *Proc. Jpn. Nurs. Assoc. (Psychiatr. Nurs.)* 2006; **37**: 119–121 (in Japanese).

Kinoshita Y, Hosoi M, Fujimoto E *et al*. A team approach to pain disorder by taking advantage of the patient's strong desire for dependence: from the stand point of the cognitive-behavioural approach by nursing staff members. *Jpn. J. Psychosom. Med.* 2007; **47**(5): 339–345 (in Japanese).

Kobayashi M. Arukouru izonshousya no tiryou no doukizuke: ninchi ryouhou-teki kakawari wo tooshite. *Jpn. J. Psychiatr. Nurs. Soc.* 2002; **45**(2): 325–329 (in Japanese).

Koga M. Ninchi koudou ryouhou wo mochiita apurouchi niyoru kouka: hi-kinouteki shikou kirokuhyou ni kansuru ichikousatsu. *Jpn. J. Psychiatr. Nurs. Soc.* 2009; **52**(2): 268–272 (in Japanese).

Koyano Y, Mori M, Tateishi A, Miyamoto M. A qualitative analysis of the process of emotional transformation for a patient with difficulty in controlling emotions: the effectiveness of the dialectical behavioural therapeutic approach of skills training in distress tolerance and mindfulness. *J. Health Care Nurs.* 2013; **10**(1): 29–37 (in Japanese).

Maekawa S. Hyoukasyakudo de shimesu ninchi ryouhou wo toriireta kango mensetsu ni yoru henka. *Seishin Kango* 2006; **9**(2): 29–35.

Masuda I, Takeda K, Matsumoto S. Ninchi no yugami ni taisuru apurouchi houhou: sukejuuruhyo wo katsuyou shi ryoukouna keika wo tadotta utsubyou kanja no ichirei. *Proc. Jpn. Nurs. Assoc. (Psychiatr. Nurs.)* 2006; **37**: 232–234 (in Japanese).

Mikami Y. The cognitive behaviour therapeutic approach as nursing intervention. *Bull. Aichi Prefect. Univ. Sch. Nurs. Health.* 2008; **14**: 105–112 (in Japanese).

Mikami Y. Mansei tougou shitchousho kanja eno byoushiki no kakutoku wo mokuteki toshita ninchi koudou ryouhou. *Seishin Kango* 2010; **13**(3): 2–32 (in Japanese).

Mikami Y, Masuda Y. Fuan wo tomonau tougou shitchoushou kanja eno kangoshi ni

yoru ninchi koudou ryouhou-teki apurouchi ni kansuru ichikousatsu. *Seishin Kango*, 2009; **12**(5): 66–74 (in Japanese).

Monden S, Nishimaru K, Kawashima C. Houmon kango ni ninnchi koudou ryouhou wo toriirete no kouka. *Jpn. J. Psychiatr. Nurs. Soc.* 2009; **52**(2): 337–341 (in Japanese).

Muto T. Konkoudansu sukiru wo mochiita kango mensetsu no kouka. *Proc. Jpn. Nurs. Assoc. (Psychiatr. Nurs.)* 2007; **38**: 81–83 (in Japanese).

Myodo F, Aida S, Ohtsuka K. Mansei tougou shitchousho kanja ni taisuru ninnchi ryouhou-teki sekkinhou: sutoresu koupingu no koujo ni panfuretto wo shiyou shite. *Proc. Jpn. Nurs. Assoc. (Psychiatr. Nurs.)* 2006; **37**: 166–168 (in Japanese).

Myojin K. Genchou ni taisuru zikotaisyo nouryoku no kakutoku ni muketa torikumi: shojo jikokanri eno kakawari. *Jpn. J. Psychiatr. Nurs. Soc.* 2007; **50**(2): 374–378 (in Japanese).

Nakata H. Adohiaransu wo kakutoku shiteiku katei eno kango kainyuu. *Jpn. J. Psychiatr. Nurs. Soc.* 2008; **51**(2): 72–75 (in Japanese).

Nobunaga K, Ichinoyama T, Sen, H. Mizuchudoku kanja no suibun sessyu houhou no kaizen ni taisuru enjo: ninchi koudou ryouhou-teki na kakawari no torikumi. *Rinsho Kango* 2010; **36**(9): 1218–1224 (in Japanese).

Ohki H, Aoyagi T. Gencho wo shushoujou tosuru tougou shitshousho kanja eno kango kainyuu: ninnchi ryouhou wo toriireta sesshon wo kokoromite. *Proc. Jpn. Nurs. Assoc. (Psychiatr. Nurs.)* 2008; **39**: 50–52 (in Japanese).

Okada Y. An approach to the cognition of woman with mild depression. *Bull. St. Lukes. Coll. Nurs.* 2005; **30**: 57–65 (in Japanese).

Okamoto T. Chuuitadousei shougaiji to kazoku ni taisuru houmon kango no torikumi: koudou ryouhou niyoru mondai koudou eno apurouchi. *Jpn. J. Psychiatr. Nurs. Soc.* 2003; **46**(2): 497–501 (in Japanese).

Okumura K. Chouki kakuri kanja no jikan syusshitsu kakudai wo mezashite: ninchi ryouhou-teki kakawari wo kokoromite no ichikousatsu. *Jpn. J. Psychiatr. Nurs. Soc.* 2007; **50**(2): 177–181 (in Japanese).

Sasaki N, Koyama N, Ota A. Seishin kango ni okeru shinri kyouiku to ninchi koudouryouhou no zissai to sono kouka: genchou/mousou kanja no ichijirei wo

tooshite. *Proc. Jpn. Nurs. Assoc. (Psychiatr. Nurs.)* 2013; **43**: 70–73 (in Japanese).

Seno Y. Shishimahi wo shushoujou to suru hisuterii kanja no kango: koudou ryouhou-teki apurouchi wo tooshite. *Proc. Jpn. Nurs. Assoc. (Pediatr. Nurs.)* 1987: 186–189 (in Japanese).

Sonehara R. Jiko hiteikan ga tsuyoi utsubyou kanja eno ninchi koudouryouhou apurouchi: fiidobakku ni mensetsunouto wo mochiita ichirei. *Jpn. J. Psychiatr. Nurs. Soc.* 2007; **50**(2): 133–137 (in Japanese).

Suzuki Y. Jisatsukito no aru utsubyou kanja no kango wo kangaeru: kazokushien wo tooshite manandakoto. *Jpn. J. Psychiatr. Nurs. Soc.* 2008; **51**(2): 426–430 (in Japanese).

Suzuki Y. Utsubyoukanja no kango wo kangaeru: kanja/kazoku eno shinrikyouiku wo kokoromite. *Jpn. J. Psychiatr. Nurs. Soc.* 2010; **53**(3): 100–104 (in Japanese).

Tadano K, Uchino T. Chouki nyuuin no tougou shitchousho kanja no kinsen jiko kanri: ninchi koudou ryouhou no ichigihou dearu mondai kaiketsuhou wo shiyoushite. *Jpn. J. Psychiatr. Nurs. Soc.* 2010; **53**(3): 85–89 (in Japanese).

Takemoto A, Fukada Y, Kato T. Bakuro hannou bougaihou wo ukeru kyouhaku shinkeisho kanja eno kango. *Jpn. J. Psychiatr. Nurs. Soc.* 2002; **45**(2): 182–186 (in Japanese).

Tanaka M. Rounenki utsubyou kanja eno ninchi ryouhou wo tooshita kakawari nitsuite: chiryou no doukizuke toshite kanja no fiidobakku eno kakawari wo tooshite. *Jpn. J. Psychiatr. Nurs. Soc.* 2007; **50**(2): 609–613 (in Japanese).

Tsuchida T, Nishimoto Y, Emoto S. Fuketsu kyouhaku shoujou wo teishita sesshokushougai kanja ni taisuru ninchi koudou ryouhou-teki taiou no yuukousei. *Proc. Jpn. Nurs. Assoc. (Psychiatr. Nurs.)* 2008; **39**: 170–172 (in Japanese).

Tsuchiya M, Ichihashi M. Koudou ryouhou ni okeru kango taiou: 17 nenkan heisai byoutou de keikashita jirei wo tooshite. *Jpn. J. Psychiatr. Nurs. Soc.* 1999; **42**(1): 219–221 (in Japanese).

Ueno K, Fukushi M, Fujii K. Summary of nursing for a patient suffered from the obsessive compulsive disorder: Respect for patient’s will and choice improves the symptom of obsession. *J. Sunagawa City Med. Cent.* 2004; **21**(1): 123–125 (in

Japanese).

Yamazaki A, Sudo Y. Kyouhakusei shougai kanja eno kango apurouchi wo kokoromite: koudouryouhou ni yoru nichijou seikatsu puroguramu jisshi kara taiin madeno keika wo tsuiseki shite. *Jpn. J. Psychiatr. Nurs. Soc.* 2002; **45**(2): 207–211 (in Japanese).

Yoshinaga N, Kobori O, Iyo M, Shimizu E. Cognitive behaviour therapy using the Clark & Wells model: a case study of a Japanese social anxiety disorder patient. *Cogn. Behav. Ther.* 2013; **6**: e3.

Yoshinaga N, Shimizu E. Social skills training encourages a patient with social anxiety disorder to undertake challenging behavioural experiments. *Br. J. Med. Med. Res.* 2014; **4**(3): 905–913.

Yuyama K. Shouni (youji/gakudou-ki) seishinka byoutou no esuesutii wo ishiki shita kangoshi no kakawari no kouka: asuperuga shougai wo motsu san jirei wo tooshite. *Proc. Jpn. Nurs. Assoc. (Pediatr. Nurs.)* 2007; **37**: 62–64 (in Japanese).
